# Uncontrolled blood pressure and its risk factors among hypertensive patients, Marrakech, Morocco

**DOI:** 10.1038/s41598-024-53115-y

**Published:** 2024-02-05

**Authors:** Safae Belayachi, Fatima Zahra Boukhari, Firdaous Essayagh, Othmane Terkiba, Ikram Marc, Abdellah Lambaki, Alban Zohoun, Meriem Essayagh, Touria Essayagh, Sanah Essayagh

**Affiliations:** 1 Faculté des Sciences et Techniques, Laboratoire Agroalimentaire et Santé, Hassan First University of Settat, Settat, Morocco; 2 Faculté des Sciences Juridiques, Économiques et Sociales, Laboratoire Droit Privé et Enjeux de Développement, Université Sidi Mohamed Ben Abdellah, Fès, Morocco; 3 Institut Supérieur des Sciences de la Santé, Laboratoire Sciences et Technologies de la Santé, Hassan First University of Settat, Settat, Morocco; 4 Faculté des Sciences de la Santé, Université de Lomé, Lomé, Togo; 5 Unité d’enseignement et de Recherche en Hématologie, Faculté des Sciences de la Santé, Cotonou, Benin; 6 Office National de Sécurité Sanitaire des Produits Alimentaires, Oriental, Morocco

**Keywords:** Epidemiology, Cardiovascular diseases

## Abstract

Hypertension is a public health problem. Failure to control blood pressure figures is responsible for morbidity and premature mortality. This study aims to describe the characteristics of hypertensive patients followed at primary health care centers in Marrakech. Between May 2021 and December 2022, a cross-sectional study of 922 hypertension patients attending primary health care centers in Marrakech was done. To gather socio-demographic, behavioral, and clinical data, as well as hypertension treatment features and the care-patient-physician triad, a face-to-face questionnaire was employed. To identify risk factors associated with uncontrolled blood pressure, multivariate logistic regression was used. Uncontrolled blood pressure was found in 73.5% of people. The patients' average age was 63.4 ± 9.4 years (mean ± standard deviation), and 524 (77.3%) were women. Tobacco consumption (Adjusted Odd Ratio of 4.34; 95% CI [1.58–11.9]); lack of self-monitoring of hypertension (AOR of 1.69; 95% CI [1.14–2.52]); a family history of hypertension (AOR of 1.58; 95% CI [1.12–2.22]); overweight or obesity (AOR of 1.73; 95% CI [1.15–2.58]); and nonadherence to antihypertensive medication (AOR of 1.58; 95% CI [1.05–2.38]) were identified as risk factors for uncontrolled blood pressure. In hypertensive individuals, the percentage of uncontrolled blood pressure is considerable. It is essential to provide therapeutic education classes for hypertension patients in order to strengthen their power and autonomy in managing their hypertension.

## Introduction

Noncommunicable diseases are responsible for 41 million deaths each year worldwide^1^. In Morocco, 80% of deaths from all causes are due to noncommunicable diseases, and 38% are related to cardiovascular diseases^2^. Hypertension, defined as systolic blood pressure (SBP) greater than or equal to 140 mmHg and/or diastolic blood pressure (DBP) greater than or equal to 90 mmHg, is the primary risk factor for cardiovascular disease^3^. According to the World Health Organization (WHO), in 2021, 1.28 billion adults between the ages of 30 and 79 suffered from hypertension^4^.

In Morocco, in 2018, the prevalence of hypertension was 29.6%^2,5^. We know that the lack of control of hypertension is responsible for cardiovascular complications such as strokes, chronic renal failure, disabilities, and early mortality. It affects the quality of life of hypertensive people. Each rise in SBP from 20 mmHg to more than 115 mmHg, or each rise in DBP from 10 mmHg to more than 75 mmHg, doubles the risk of developing cardiovascular events and major strokes^6^. The management of hypertension aims to ensure the control of blood pressure and the reduction of cardiovascular risks, morbidity, and mortality. Clinical trials have indicated that antihypertensive medications lower the risk of stroke by 35–40%, myocardial infarction by 15–25%, and heart failure by 64%^7–9^. In 2017, 73% of hypertensive people in Meknes, Morocco, had uncontrolled blood pressure (UBP)^10^. The lack of control over hypertension is an obstacle to achieving sustainable development goals in terms of health. The objective of our study was this study aims to describe the characteristics of hypertensive patients followed at primary health care centers in Marrakech and identify the associated risk factors of UBP.

## Methods

Marrakech is a city located in the center of Morocco. It covers an area of approximately 230 km^2^ and has a population of 1,330,468 people^11^, including 23,213 hypertensives treated by the 79 primary health care centers in Marrakech^12^. A cross-sectional study was conducted between May 2021 and December 2022 among hypertensive patients followed at primary health care centers in Marrakech. We stratified by area of residence since statistics from the Marrakech prefecture's health delegation show that 70% of hypertension patients live in urban areas and 30% live in rural areas. An exhaustive list of primary health care centers was provided by the delegation of the Ministry of Health of Marrakech. Data collection was made by convenience sampling. A first draw was carried out to select 15 health centers among the 79 health care centers present in Marrakech. A second draw was made to determine the hypertensive patients aged 18 years or older who were present at primary health care centers on the day of the survey and agreed to participate in the study. To determine hypertensive patients in each selected center, hypertensive patients were assigned a queue number upon arrival, and these numbers were randomly drawn to select patients until the required sample size was reached.

### Inclusion criteria

We included in the study participants who were previously known to be hypertensive due to a medical diagnosis, were on antihypertensive treatment for at least six months, were monitored at primary health care centers in Marrakech, and consented to participate in the study.

### Exclusion criteria

We excluded pregnant women and patients with mental disorders from the study.

### Data collection tool and procedure

Data collection was carried out through a questionnaire administered face-to-face after an interview with patients. This questionnaire contained sociodemographic and economic characteristics, behavioral characteristics, knowledge of hypertension, clinical characteristics, characteristics of antihypertensive treatment, and the relationship between the care system, patient, and physician.

### Operational definitions

#### Uncontrolled blood pressure

Blood pressure was taken on both arms at the end of the interview using an electronic tensiometer (Microlife). Blood pressure was taken in a seated position, back leaning against the chair, feet flat on the floor, and arm at heart level. Two other measurements spaced two minutes apart were taken in the arm where the blood pressure was the highest. The blood pressure taken was the average of the last three measurements. The classification of blood pressure was made according to the recommendations of the European Society of Hypertension and the European Society of Cardiology (ESH/ESC)^13^. Hypertension was considered uncontrolled when SBP was greater than or equal to 140 mmHg and/or DBP was greater than or equal to 90 mmHg in the general population. In people with diabetes, we spoke of UBP when the SBP was greater than or equal to 140 mmHg and/or the DBP was greater than or equal to 85 mmHg. For people with renal insufficiency, we spoke of UBP when the SBP was greater than or equal to 130 mmHg and/or the DBP was greater than or equal to 90 mmHg^13^.

#### General knowledge about high blood pressure

General knowledge of high blood pressure has included symptoms (headaches, whistling, visual disturbances, dizziness, palpitations, difficulty breathing, epistaxis, hematuria), complications (heart attack, kidney damage, eye damage, and cerebrovascular accident), and preventive measures (hygiene-dietary, treatment adherence, self-measurement, and regular medical monitoring). General knowledge of high blood pressure was deemed satisfactory if the patient recognized at least half of the factors related to symptoms, complications, and prevention; otherwise, it was deemed unsatisfactory.

#### Drug non-adherence

Drug non-adherence was assessed using the Girerd test^5,14^. This test contains six questions to which the participant must answer "yes" or "no." “Yes” has a value of 1 point, and “no” has a value of 0 points. Adding the points for each question gives a score between 0 and 6. When the score is greater than or equal to one, we are talking about non-adherence to antihypertensive medication. Otherwise, we talk about good adherence. These questions are: did you forget to take your medication this morning? Since the last consultation, have you run out of medication? Have you ever taken your treatment later than usual? Have you ever not taken your medication because some days your memory fails you? Have you ever not taken your medication because some days you feel like your medication is doing more harm than good? Do you think you have too many tablets to take?.

#### Other variables

Anthropometric measurements included weight (in kilograms) and height (in meters). The body mass index (BMI) defined as the ratio between weight per kilogram and height per square meter, was classified into two categories: absence of overweight or obesity when the BMI was less than 25 kg per square meter and presence of overweight or obesity when the BMI was greater than or equal to 25 kg per square meter^15,16^.

- A tobacco user was defined as anyone who has used tobacco in the last three months.
- An alcohol user was defined as anyone who consumed alcohol in the past three months.
- The presence of comorbidity was defined as anyone with diabetes, dyslipidemia, or renal failure.
- The presence of a cardiovascular complication was defined as the presence of a self-reported history of myocardial infarction, stroke, stenting, angioplasty, or coronary artery bypass grafting.

### Ethical consideration

The study complied with the Declaration of Helsinki. Informed consent from all participants was obtained after informing them about the aims of the research and respecting their privacy and confidentiality. The Ethics Committee of the Faculty of Medicine and Pharmacy of Rabat in Morocco read, reviewed, and approved the study protocol.

### Statistical analysis

All the data was entered in Excel and analyzed on Epi-Info version 7. A descriptive analysis of the entire study population was carried out. Continuous variables were expressed as the mean and standard deviation, if appropriate. Categorical variables were expressed as numbers and percentages, where appropriate. Continuous variables were compared using the Analysis Variances test, if appropriate. Categorical variables were compared using the Pearson chi-square test when appropriate. During the bivariate analysis, all variables with a p-value up to 0.20 were included in the multiple logistic regression. The association between the risk factor and the presence of UBP was determined by the adjusted odds ratio (AOR) and its 95% confidence interval.

## Results

### Socio-demographic and economic characteristics

During the study period, a total of 922 participants were collected, with a percentage of UBP of 73.5%. The average age of the participants was 63.1 ± 9.8 years, with extremes ranging from 33 to 102 years. 727 (78.8%) were women, and 649 (70.4%) had a monthly income per household of less than $200 (Table 1).

**Table 1:** Socio-demographic and economic characteristics of hypertensive patients followed in primary health care centers, Marrakech, 2021–2022.

	Total participants n (%)	Uncontrolled blood pressure n (%)	Controlled blood pressure n (%)	*p*-value
Total participants	922 (100)	678 (73.5)	244 (26.5)	
Mean age in year ± sd	63.1 ± 9.8	63.4 ± 9.4	62.3 ± 10.7	0.11
Sex				
Female	727 (78.8)	524 (77.3)	203 (83.2)	0.05
Male	195 (21.2)	154 (22.7)	41 (16.8)	
Age group in years				
80 and older	54 (05.9)	39 (05.8)	15 (06.1)	0.11
70–79	182 (19.7)	135 (19.9)	47 (19.2)	
60–69	386 (41.9)	298 (43.9)	88 (36.1)	
50–59	225 (24.4)	158 (23.3)	67 (27.5)	
Less than or equal to 49	75 (08.1)	48 (07.1)	27 (11.1)	
Marital status				
Single	346 (37.5)	262 (38.6)	84 (34.4)	0.24
Partnered	576 (62.5)	416 (61.4)	160 (65.6)	
Education				
Illiterate	690 (74.8)	508 (74.9)	182 (74.6)	0.78
Elementary	151 (16.4)	111 (16.4)	40 (16.4)	
Middle school	47 (05.1)	36 (05.3)	11 (04.5)	
High school	21 (02.3)	13 (01.9)	8 (03.3)	
College	13 (01.4)	10 (01.5)	3 (01.2)	
Occupation				
No	833 (90.4)	609 (89.8)	224 (91.8)	0.36
Yes	89 (09.6)	69 (10.2)	20 (08.2)	
Monthly income per household ($)				
< 150	281 (30.5)	225 (33.2)	56 (22.9)	0.04
150–199	368 (39.9)	262 (38.6)	106 (43.4)	
200–299	235 (25.5)	166 (24.5)	69 (28.3)	
300–499	23 (02.5)	15 (02.2)	8 (03.3)	
≥ 500	15 (01.6)	10 (01.5)	5 (02.1)	
Health insurance				
No	156 (16.9)	120 (17.7)	36 (14.8)	0.29
Yes	766 (83.1)	558 (82.3)	208 (85.2)	

*Sd.* standard deviation.

### Data on knowledge of hypertension and behavioral characteristics

Table 2 summarizes the data in relation to knowledge about hypertension and the behavioral characteristics of the participants. Thus, out of 922 participants, 905 (98.2%) had unsatisfactory general knowledge about hypertension, and 912 (98.9%) had unsatisfactory knowledge about its preventive measures. A total of 86 (09.3%) were tobacco users, and 739 (80.1%) did not self-monitor their blood pressure at home.

**Table 2:** Data on knowledge about hypertension and behavioral characteristics of hypertensive patients followed at primary health care centers, Marrakech, 2021–2022.

	Total participants n (%)	Uncontrolled blood pressure n (%)	Controlled blood pressure n (%)	*p*-value
General knowledge about hypertension				
Unsatisfactory	905 (98.2)	671 (99.0)	234 (95.9)	0.004
Satisfactory	17 (1.8)	7 (01.0)	10 (04.1)	
Knowledge of hypertension signs				
Unsatisfactory	904 (98.1)	667 (98.4)	237 (97.1)	0.22
Satisfactory	18 (01.9)	11 (01.6)	7 (02.9)	
Knowledge of hypertension complications				
Unsatisfactory	592 (64.2)	434 (64.0)	158 (64.7)	0.83
Satisfactory	330 (35.8)	244 (36.0)	86 (35.3)	
Knowledge of hypertension preventive measures				
Unsatisfactory	912 (98.9)	676 (99.7)	236 (96.7)	0.002
Satisfactory	10 (01.1)	2 (00.3)	8 (03.3)	
Tobacco consumption				
Yes	86 (09.3)	78 (11.5)	8 (03.3)	0.0004
No	836 (90.7)	600 (88.5)	236 (96.7)	
Alcohol consumption				
Yes	39 (04.2)	36 (05.3)	3 (01.2)	0.01
No	883 (95.8)	642 (94.7)	241 (98.8)	
Stress				
Intense	229 (24.8)	171 (25.2)	58 (23.8)	0.53
Moderate	586 (63.6)	433 (63.9)	153 (62.7)	
Low	107 (11.6)	74 (10.9)	33 (13.5)	
Physical activity				
Unsatisfactory	641 (69.5)	486 (71.7)	155 (63.5)	0.06
Satisfactory	281 (30.5)	192 (28.3)	89 (36.5)	
Salty diet				
Salty	253 (27.4)	192 (28.3)	61 (25.0)	0.31
Semi-salty	669 (72.6)	486 (71.7)	183 (75.0)	
Consumption of five fruits and vegetables per day				
No	486 (52.7)	374 (55.2)	112 (45.9)	0.01
Yes	436 (47.3)	304 (44.8)	132 (54.1)	
Self-monitoring of hypertension				
No	739 (80.1)	559 (82.5)	180 (73.8)	0.003
Yes	183 (19.9)	119 (17.5)	64 (26.2)	
Social support				
No	351 (38.1)	275 (40.6)	76 (31.1)	0.009
Yes	571 (61.9)	403 (59.4)	168 (68.9)	

### Clinical characteristics

**Figure 1:**
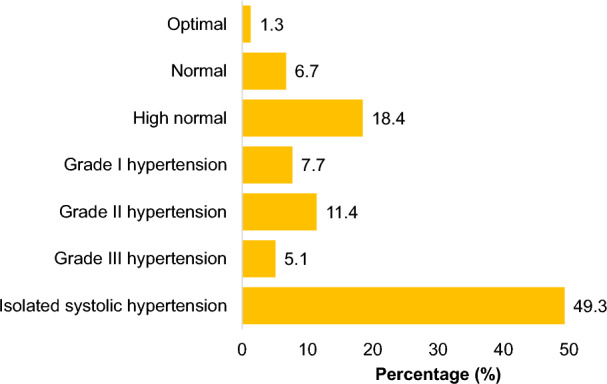
Blood pressure classification of hypertensive patients followed at primary health care centers in Marrakech, 2021–2022.

The data in Table 3 shows that 464 (50.3%) of participants had comorbidities, 418 (45.3%) had diabetes, 542 (58.8%) had a family history of hypertension, 742 (80.5%) were overweight or obese, 551 (59.8%) reported symptoms of depression, and 319 (34.6%) reported symptoms of anxiety. The mean systolic blood pressure (SBP) was 151.4 ± 18.1 mmHg, while the diastolic blood pressure was 81.9 ± 11.0 mmHg in the hypertensive patients. Isolated arterial hypertension was present in 455 (49.3%) of the cases (Fig. 1).

**Table 3:** Clinical characteristics of hypertensive patients followed at primary health care centers in Marrakech, 2021–2022.

	Total participants n (%)	Uncontrolled blood pressure n (%)	Controlled blood pressure n (%)	*p*-value
Mean systolic blood pressure in mmHg ± sd	151.4 ± 18.1	158.4 ± 15.6	131.7 ± 6.2	
Mean diastolic blood pressure in mmHg ± sd	81.9 ± 11.0	84.9 ± 10.7	73.7 ± 7.1	
Comorbidity				
Yes	464 (50.3)	364 (53.7)	100 (41.0)	0.0007
No	458 (49.7)	314 (46.3)	144 (59.0)	
Diabetes				
Yes	418 (45.3)	332 (49.0)	86 (35.2)	0.0002
No	504 (54.7)	346 (51.0)	158 (64.8)	
Dyslipidemia				
Yes	54 (05.9)	37 (05.5)	17 (07.0)	0.38
No	868 (94.1)	641 (94.5)	227 (93.0)	
Presence of hypertension complications				
Yes	60 (06.5)	44 (06.5)	16 (06.6)	ND
No	862 (93.5)	634 (93.5)	228 (93.4)	
Duration of hypertension in years				
> 3	638 (69.2)	481 (70.9)	157 (64.3)	0.05
≤ 3	284 (30.8)	197 (29.1)	87 (35.7)	
Family history of hypertension				
Yes	542 (58.8)	411 (60.6)	131 (53.7)	0.06
No	380 (41.2)	267 (39.4)	113 (46.3)	
Depression symptoms				
Yes	551 (59.8)	423 (62.4)	128 (52.5)	0.006
No	371 (40.2)	255 (37.6)	116 (47.5)	
Anxiety symptoms				
Yes	319 (34.6)	248 (36.6)	71 (29.1)	0.03
No	603 (65.4)	430 (63.4)	173 (70.9)	
Overweight/obesity				
Yes	742 (80.5)	560 (82.6)	182 (74.6)	0.007
No	180 (19.5)	118 (17.4)	62 (25.4)

### Characteristics related to treatment and the patient-physician-Healthcare system triad

A total of 776 (84.2%) participants reported an unsatisfactory relationship with the healthcare system, 778 (84.4%) had an unsatisfactory relationship with their physician, and 760 (82.4%) had drug nonadherence (Table 4).

**Table 4:** Data on antihypertensive treatment and characteristics related to the care-patient-physician system in hypertensive patients followed at primary health care centers, Marrakech, 2021–2022.

	Total participants n (%)	Uncontrolled blood pressure n (%)	Controlled blood pressure n (%)	*p*-value
Patient-care system relationship				
Unsatisfactory	776 (84.2)	583 (86.0)	193 (79.1)	0.01
Satisfactory	146 (15.8)	95 (14.0)	51 (20.9)	
Patient-physician relationship				
Unsatisfactory	778 (84.4)	584 (86.1)	194 (79.5)	0.01
Satisfactory	144 (15.6)	94 (13.9)	50 (20.5)	
Type of treatment				
Tripletherapy	878 (95.2)	641 (94.5)	237 (97.1)	0.21
Bitherapy	38 (04.1)	31 (04.6)	7 (02.9)	
Monotherapy	6 (00.7)	6 (00.9)	0 (00.0)	
Duration of treatment in months				
Greater than six	576 (62.5)	429 (63.3)	147 (60.2)	0.40
Less than or equal to six	346 (37.5)	249 (36.7)	97 (39.8)	
Adherence to antihypertensive drugs				
No	760 (82.4)	576 (85.0)	184 (75.4)	0.001
Yes	162 (17.6)	102 (15.0)	60 (24.6)	

### Uncontrolled blood pressure

A total of 678 participants had UBP, which means a percentage of 73.5%. Their average age was 63.4 ± 9.4 years, 524 (77.3%) were women, and 487 (71.8%) had a monthly income per household of less than 200 dollars (Table 1). 78 (11.5%) participants used tobacco, and 559 (82.5%) declared not self-monitoring their blood pressure at home (Table 2). 560 (82.6%) were overweight or obese, and 411 (60.6%) had a family history of hypertension (Table 3). The mean systolic blood pressure (SBP) was 158.4 ± 15.6 mmHg, while the diastolic blood pressure was 84.9 ± 10.7 mmHg. The unsatisfactory relationship with the healthcare system was observed in 583 (86.0%) participants, and non-adherence to antihypertensive drugs in 576 (85.0%) (Table 4).

As mentioned in Table 5, after bivariate analysis, the following variables were associated with the presence of poor hypertension control: 1. female sex; 2. age; 3. low monthly income per household; 4. unsatisfactory knowledge about hypertension; 5. unsatisfactory knowledge about preventive measures against hypertension; 6. tobacco consumption; 7. alcohol consumption; 8. low- to moderate-intensity physical activity; 9. non-consumption of five fruits and vegetables per day; 10. lack of self-monitoring; 11. lack of family support; 12. presence of comorbidity; 13. presence of diabetes; 14. duration of hypertension of more than three years; 15. presence of a family history of hypertension; 16. presence of self-reported symptoms of depression; 17. presence of self-reported anxiety symptoms; 18. presence of overweight or obesity; 19. unsatisfactory relationship between patient and healthcare system; 20. unsatisfactory patient-physician relationship; and 21. non-adherence to antihypertensive medication.

**Table 5:** Multivariate analysis (odds ratio, p-value) of risk factors associated with uncontrolled blood pressure among hypertensive patients, Marrakech, Morocco, 2021–2022.

	Bivariate analysis		Multivariate analysis complete model	
	COR (95% CI)	*p*-value	AOR (95% CI)	*p*-value
Female sex	0.68 [0.46–1.00]	0.05	0.79 [0.48–1.30]	0.36
Age group in years		0.11		
≥ 80/ ≤ 49	1.46 [0.68–3.12]	0.32	1.57 [0.64–3.82]	0.31
70–79/ ≤ 49	1.61 [0.90–2.87]	0.10	1.22 [0.63–2.38]	0.54
60–69/ ≤ 49	1.90 [1.12–3.23]	0.01	1.64 [0.90–2.97]	0.10
50–59/ ≤ 49	1.32 [0.76–2.30]	0.31	1.21 [0.66–2.20]	0.52
Monthly income in dollars ($)		0.04		
< 150/ ≥ 500	2.00 [0.66–6.11]	0.21	0.91 [0.23–3.50]	0.89
150–199/ ≥ 500	1.23 [0.41–3.70]	0.70	0.68 [0.18–2.56]	0.57
200–299/ ≥ 500	1.20 [0.39–3.64]	0.74	0.73 [0.19–2.74]	0.64
300–499/ ≥ 500	0.93 [0.23–3.70]	0.92	0.56 [0.11–2.68]	0.47
Insufficient general knowledge about hypertension	4.09 [1.54–10.8]	0.004	0.63 [0.10–3.83]	0.62
Insufficient knowledge about preventive measures against hypertension	11.4 [2.41–54.2]	0.002	10.22 [0.80–129.3]	0.07
Tobacco consumption	3.83 [1.82–8.05]	0.0004	4.34 [1.58–11.9]	0.004
Alcohol consumption	4.50 [1.37–14.7]	0.01	1.40 [0.31–6.21]	0.65
Physical activity		0.06		
Intense/low	1.52 [0.85–2.72]	0.15	1.31 [0.68–2.54]	0.41
Moderate/low	1.06 [0.57–1.97]	0.84	1.07 [0.53–2.15]	0.83
Not consuming five vegetables and fruit every day	1.44 [1.08–1.94]	0.01	1.11 [0.80–1.54]	0.50
Lack of self-monitoring	1.67 [1.18–2.36]	0.003	1.69 [1.14–2.52]	0.008
Lack of family support	1.50 [1.10–2.05]	0.009	1.21 [0.85–1.73]	0.28
Presence of comorbidities	1.66 [1.24–2.24]	0.0007	0.91 [0.45–1.85]	0.81
Presence of diabetes	1.76 [1.30–2.38]	0.0002	1.89 [0.93–3.84]	0.07
Duration of hypertension more than 3 years	1.35 [0.99–1.84]	0.05	1.17 [0.82–1.66]	0.36
Presence of history of hypertension	1.32 [0.98–1.78]	0.06	1.58 [1.12–2.22]	0.007
Depression symptoms	1.50 [1.11–2.02]	0.006	1.11 [0.76–1.61]	0.58
Anxiety symptoms	1.40 [1.02–1.93]	0.03	0.98 [0.66–1.46]	0.95
Overweight or obesity	1.61 [1.13–2.29]	0.007	1.73 [1.15–2.58]	0.007
Unsatisfactory patient-healthcare-system relationship	1.62 [1.11–2.36]	0.01	1.46 [0.97–2.22]	0.06
Unsatisfactory patient-physician relationship	1.60 [1.09–2.34]	0.01	1.28 [0.83–1.97]	0.26
Non adherence to hypertensive drugs	1.84 [1.28–2.63]	0.001	1.58 [1.05–2.38]	0.02

### Multivariate analysis

After controlling for the other variables, we identified the following factors as associated with poor hypertension control: 1. tobacco consumption (Adjusted Odd Ratio of 4.34; 95% CI [1.58–11.9]); 2. lack of self-monitoring (AOR of 1.69; 95% CI [1.14–2.52]); 3. presence of a family history of hypertension (AOR of 1.58; 95% CI [1.12–2.22]); 4. presence of overweight or obesity (AOR of 1.73; 95% CI [1.15–2.58]); and 5. non-adherence to antihypertensive drugs (AOR of 1.58; 95% CI [1.05–2.38]) (Table 5).

## Discussion

Our study estimated the percentage of UBP at 73.5% among hypertensives followed at primary health care centers in Marrakech. This result is similar to the 73.0% observed in Meknes in 2017 in 922 hypertensive patients^10^. In developing countries, the percentage of poor hypertension control varies between 69 and 77%^17,18^. Indeed, in 12 sub-Saharan countries, among 2198 hypertensive patients, the percentage of poor hypertension control was 77.4%^17^. In Turkey, in 2017, out of 556 hypertensive patients, the percentage of poor hypertension control was 69.8%^18^. In Ghana, in 2018, out of 2870 hypertensive patients, the percentage of poor hypertension control was 57.7%^19^. In developed countries, this percentage is between 50 and 61%. Thus, in the United Kingdom, out of 100,000 hypertensive patients, 61.9% had UBP^20^, and in the United States, between 2011 and 2014, in 9623 hypertensives, the percentage of UBP was 53.4%^21^.

In our study, tobacco consumption was associated with UBP. Several studies have reported tobacco as a risk factor associated with the UBP^22–24^. Tobacco includes nicotine, which causes vasoconstriction of blood vessels^25^. It also activates the sympathetic nervous system, producing an increase in heart rate and blood pressure^26^. People who smoke can have damaged blood vessels, causing the formation of plaques in the arteries^27^. This narrowing creates a significant resistance to blood flow, thus causing an increase in blood pressure.

Self-monitoring of blood pressure with accurate electronic blood pressure monitors is becoming increasingly important in the treatment of hypertension^28^. It allows better control of blood pressure and a reduction in cardiovascular complications^29^. It also reduces the white coat effect and ensures the availability of blood pressure data in different places and at different times. Blood pressure self-monitoring is a form of self-management that involves and empowers individuals in the treatment of their condition. It represents a source of motivation for therapeutic adherence and a way of evaluating the effectiveness of antihypertensive treatment. In our study, the absence of self-monitoring was associated with UBP.

As in our survey, several other studies have demonstrated that there is an important relationship between the presence of a family history of hypertension and UBP^10^. His relationship can be explained by genetics and heritability^30^, adoption of an unhealthy lifestyle, smoking and physical inactivity^31^.

In our study, overweight and obesity were associated with UBP. The literature reports an increase in the activity of the sympathetic nervous system in obese subjects^32^. This activity causes arterial vasoconstriction and decreased renal perfusion. As a result, the renin-angiotensin system is activated, which also produces sodium and water retention, thus promoting an increase in blood pressure^33^. In 2003, a meta-analysis of 4874 hypertensive patients showed that reducing body weight leads to a decrease in blood pressure. For every kilogram of weight lost, systolic and diastolic blood pressure decrease by approximately 1 mmHg^34^.

In our study, non-adherence to antihypertensive drugs was associated with UBP. This same result was observed in other studies^5,10,35^. This could be explained by a lack of knowledge about the adverse effects of antihypertensive drugs, repeated stock-outs of antihypertensive drugs at primary health care centers, high cost, low monthly household income, and a lack of social support.

## Limits of the study

Our study has certain limitations, such as the social desirability bias seen while gathering data on alcohol and tobacco intake, as well as the willful lying bias experienced when gathering data on monthly income.

## Conclusion

The percentage of people with hypertension who have UBP is significant. Smoking, being overweight or obese, having a family history of hypertension, a lack of self-monitoring of blood pressure, and nonadherence to antihypertensive medication were all risk factors for UBP. Therapeutic education and patient empowerment in disease management are important.

## Data Availability

All data generated or analyzed during this study are included in this published article.
